# Physicochemical and antibacterial evaluation of novel nano α-TCP–AgNPs biocomposites for direct pulp-capping applications

**DOI:** 10.3389/froh.2025.1710351

**Published:** 2026-01-06

**Authors:** Selviana Wulansari, Hendra Dian Adhita Dharsono, Nasrul Wathoni, Rosalina Tjandrawinata, Arief Cahyanto, Moehamad Orliando Roeslan

**Affiliations:** 1 Doctoral Program, Faculty of Dentistry, Universitas Padjadjaran, Sumedang, West Java, Indonesia; 2 Department of Conservative Dentistry, Faculty of Dentistry, Universitas Trisakti, Jakarta, Indonesia; 3 Department of Conservative Dentistry, Faculty of Dentistry, Universitas Padjadjaran, Sumedang, West Java, Indonesia; 4 Department of Pharmaceutics and Pharmaceutical Technology, Universitas Padjadjaran, Sumedang, West Java, Indonesia; 5 Department of Dental Materials, Faculty of Dentistry, Universitas Trisakti, Jakarta, Indonesia; 6 ⁠Department of Clinical Sciences, College of Dentistry, Ajman University, Ajman, United Arab Emirates; 7 ⁠Centre of Medical and Bio-allied Health Sciences Research, Ajman University, Ajman, United Arab Emirates; 8 Department of Oral Biology, Faculty of Dentistry, Universitas Trisakti, Jakarta, Indonesia

**Keywords:** nano α-tricalcium phosphate, silver nanoparticles, pulp capping, bioactive material, ion release, antibacterial activity

## Abstract

**Background:**

Nano α-tricalcium phosphate (nano α-TCP) and silver nanoparticles (AgNPs) both possess bioactive qualities needed for pulp-capping materials. AgNPs effectively combat microbial infections without sacrificing cell viability, while nano α-TCP releases calcium and phosphate ions necessary for dentin regeneration. To assess the connection between composite structure and function, a thorough characterization utilizing Fourier transform infrared spectroscopy (FTIR), X-ray diffraction (XRD), scanning electron microscopy (SEM), ion-release analysis, and pH measurements is necessary.

**Purpose:**

This study aimed to develop and evaluate a novel biocomposite composed of nano α-TCP and AgNPs for application in direct pulp capping. The investigation focused on the material's chemistry and physical properties, as well as its antibacterial activity against *Streptococcus mutans* and *Lactobacillus acidophilus*.

**Methods:**

We prepared combinations of nano α-TCP and AgNPs with varying concentrations of AgNPs. Subsequently, we analyzed the composites using XRD, FTIR, and SEM. The investigation also evaluated pH stability and the release of ions (Ca²⁺ and PO₄³⁺) at 1, 3, 72, and 504 h. Antibacterial assays were performed against *Streptococcus mutans* and *Lactobacillus acidophilus.*

**Results:**

FTIR and XRD analyses confirmed that the functional groups and crystallinity in the nano α-TCP matrix remained unchanged following incorporation of AgNPs. SEM imaging demonstrated that the particles were uniformly distributed with minimal agglomeration. The 1% AgNPs concentration showed the best results, with a steady release of ions, a stable alkaline pH that helped with mineralization, good mechanical strength (according to ISO 9917-1), and strong antibacterial activity. The 1% AgNPs concentration also had the most potent antibacterial effect.

**Conclusion:**

The nano α-TCP/AgNPs composites, particularly the 1% AgNPs formulation, exhibited sustained calcium and phosphate ion release, stable alkaline pH, adequate compressive strength, and strong *in vitro* antibacterial activity against *Streptococcus mutans* and *Lactobacillus acidophilus*. These physicochemical and antibacterial properties highlight that this biocomposite is a promising candidate for future direct pulp-capping applications. However, its cellular responses and dentin regenerative potential must be confirmed through dedicated *in vitro* and *in vivo* studies.

## 1 Introduction

Direct pulp capping and other conservative methods to protect the tooth pulp are vital parts of the expanding field of regenerative endodontics. Traditionally, calcium hydroxide and mineral trioxide aggregate (MTA) have been the main treatments (1). However, these materials cause several problems, as they are weak, release ions at irregular rates, have poor sealing ability, and are inefficient against bacteria. These limitations make long-term treatment less successful, especially when both infection control and biological stimulation are required (2).

As a result, an increasing amount of research had focused on modifying pulp-capping agents by incorporating additional bioactive components. Nanomaterial-based pulp-capping agents can promote odontogenic differentiation, provide long-lasting antibacterial effects, regulate the physicochemical microenvironment, and maintain their form and structure even under functional stress (3). In this case, Fourier transform infrared spectroscopy (FTIR) provides insight into composition and chemical bonding, X-ray diffraction (XRD) reveals changes in structure and crystallinity, and scanning electron microscopy (SEM) enables visualization of the material's surface and structure (4, 5).

Nano α-tricalcium phosphate (nano α-TCP) is a biodegradable and bioactive calcium phosphate ceramic that is very similar to the minerals found in human hard tissues like bone and dentin (6, 7). It is particularly significant for regenerative dental applications as it can transform into hydroxyapatite (HAp). Nano α-TCP has a substantially larger specific surface area and dissolves more readily when manufactured at the nanoscale. These properties increase its reactivity, enabling swiftly ion exchange with nearby biological fluids (8).

These structures allow calcium (Ca^2+^) and phosphate (PO_4_^3−^) ions, which are vital for coordinating the healing responses of the pulp, to be released in a steady and controlled manner. Ca^2+^ ions not only assist mineral development by functioning as building blocks but also provide signals inside cells that affect gene expression related to odontoblastic differentiation and dentinogenesis (9). PO_4_^3−^ ions support this process by providing places in the extracellular matrix where mineral crystals can grow. This dual-ion synergy creates a bioactive environment that helps build dentin bridges, maintains a stable collagen matrix, and keeps the pulp alive for an extended period (10). Therefore, nano α-TCP is an excellent choice for pulp therapy materials, particularly when combined with additional agents like silver nanoparticles (AgNPs) to provide antibacterial effects to regenerative endodontics (11).

Silver nanoparticles (AgNPs) have been widely used in various biomedical applications due to their antimicrobial properties and high biological activity. However, the cytotoxicity of AgNPs toward human cells remains a major concern. A recent study by Konappa et al. (12) demonstrated that green-synthesized AgNPs using Amomum nilgiricum leaf extract were safe for human cells at concentrations below 10 µg/mL, without triggering significant apoptotic activity. At concentrations above 25 µg/mL, however, there was increased caspase-3 and caspase-8 expression, indicating the activation of the cell death pathway through apoptosis (12).

AgNPs are also well known for their strong broad-spectrum antibacterial activity. They achieve this in several ways, including breaking down the integrity of bacterial cell membranes, interfering with enzymatic activity, and generating reactive oxygen species (ROS) that induce oxidative stress. These nanoparticles can bind to thiol groups in bacterial proteins, modify the structure of key enzymes, and inhibit DNA replication. This will ultimately kill the bacteria. As AgNPs increase in nano size, their surface area changes, which enhances their ability to interact with microbial membranes and improves their performance even when present in low concentrations (11).

When combined with a calcium phosphate matrix, such as nano α-TCP, AgNPs can be evenly distributed and released slowly. This stops them from destroying cells while still killing bacteria. This integration not only provides the tissue with a place to grow but also protects the pulp from pathogens (13). AgNPs may also affect how the immune system functions and how cells communicate with one another, whether they are healing or deteriorating. AgNPs play a crucial role in the design of next-generation biomaterials for pulp-capping applications, as they can kill pathogens and modulate the body's response. These products are used to both control infections and speed up healing (14).

The regenerative characteristics of nano α-TCP underscore the importance of effective management of bacterial contamination at the exposure site, which remains a significant challenge for clinical outcomes. AgNPs exhibit broad-spectrum antibacterial activity against common oral pathogens, including *Streptococcus mutans* and *Lactobacillus acidophilus*, which are often associated with endodontic issues (15). When incorporated into a nano α-TCP matrix at controlled low concentrations, AgNPs can significantly compromise bacterial membranes and inhibit DNA replication while maintaining host cell viability (16). The strategic combination of AgNPs and nano α-TCP creates a dual-functional pulp-capping material that promotes healing procedures and acts as an antibacterial barrier, thereby enhancing the overall efficiency of vital pulp therapy (17).

Understanding how AgNPs affect the physicochemical behavior of nano α-TCP is crucial for optimizing both regeneration and antibacterial efficacy. This requires a systematic evaluation of properties that drive biological responses, including ion-release kinetics, pH stability, crystallinity, and surface morphology. To our knowledge, no previous study has investigated the interaction between nano α-TCP and AgNPs in a pulp-capping composite using a combined approach of FTIR, XRD, SEM, ion-release, and pH analyses. This work addresses that gap by linking structural and chemical data to antibacterial performance.

Deep dentinal caries frequently results in pulp exposure, and contemporary caries management emphasizes minimally invasive vital pulp therapies rather than extraction or root canal treatment (1, 2). In this context, a pulp-capping material that simultaneously suppresses cariogenic microbiota and supports hard-tissue repair directly contributes to dental caries prevention and management. The present nano α-TCP/AgNPs biocomposites were therefore designed to combine sustained alkaline pH and Ca^2+^/PO₄³^−^ release with targeted antibacterial activity against *S. mutans* and *L. acidophilus*, two major species implicated in caries progression. By stabilizing the pulp–dentin interface beneath deep carious lesions, such materials may help arrest disease activity, maintain tooth vitality, and reduce the need for more invasive endodontic procedures.

## 2 Materials and methods

### 2.1 Materials preparation

Alpha-tricalcium phosphate (α-TCP, HiMedia, Mumbai, India) powder was synthesized by dry high-energy ball milling (HEM) to produce nanoparticles. The process was carried out in a planetary ball mill with zirconia balls 2–5 mm in diameter as the grinding media inside a zirconia jar to avoid metal contamination. The ball-to-powder ratio (BPR) was set at 10:1, with a rotation speed of 400 rpm for 5 effective hours using a duty cycle of 15 min ON and 5 min OFF to prevent excessive temperature rise (maximum < 60°C). The entire process was carried out under dry conditions at room temperature. Silver nanoparticles (AgNPs, Nanografi, Ankara, Turkey) with particle sizes <100 nm were purchased commercially and incorporated into the nano α-TCP matrix. The mixing was performed using a clean paper substrate to pre-blend the powders, which were then placed into an amalgamator capsule and agitated at high speed to achieve a homogeneous dispersion of AgNPs within the nano α-TCP matrix. Composite powders were prepared at various AgNPs concentrations to evaluate the effect of silver content on material characteristics and bioactivity (1%, 5%, and 10% w/w) (Table 1). The biocomposite powders were then incorporated into a resin matrix composed of UDMA (Sigma Aldrich, St. Louis, USA) and TEGDMA (Sigma Aldrich, St. Louis, USA) in a 1:1 ratio, silanized using 3-methacryloxypropyltrimethoxysilane (Sigma Aldrich, St. Louis, USA), and light-cured using a blue LED unit (Demi Plus, Kerr, CA, USA) (wavelength: 430–480 nm) for 20 s (18).

**Table 1:** Composition of the experimental nano α-TCP/AgNPs composites and control material. The total filler for the nano α-TCP/AgNPs composites is 60 wt% (1.8 g), while the resin matrix is 40 wt% (1.2 g).

Group	Nano α-TCP (wt%, g)	AgNPs (wt%, g)	UDMA (wt%, g)	TEGDMA (wt%, g)	Silane (wt%, g)	Camphorquinone (wt%, g)	Description
A	90; 1.62	10; 0.18	65; 0.78	35; 0.42	7; 0.21	0.7; 0.021	Nano α-TCP + 10% AgNPs
B	95; 1.71	5; 0.09	65; 0.78	35; 0.42	7; 0.21	0.7; 0.021	Nano α-TCP + 5% AgNPs
C	99; 1.78	1; 0.02	65; 0.78	35; 0.42	7; 0.21	0.7; 0.021	Nano α-TCP + 1% AgNPs
D	100; 1.80	0; –	65; 0.78	35; 0.42	7; 0.21	0.7; 0.021	Nano α-TCP without AgNPs
E	N/A	N/A	N/A	N/A	N/A	N/A	TG CaviLiner (commercial positive control; composition per manufacturer)

After weighing all the materials using a digital analytical balance, the powder was mixed and stirred using a spatula. It was then mixed with 0.21 g of silane, followed by 0.78 g of UDMA liquid and 0.42 g of TEGDMA. All materials were weighed using a digital analytical balance, and the liquids were mixed on a paper pad. Then, 0.021 g of camphorquinone powder (Sigma-Aldrich, St. Louis, USA) was added and stirred until homogeneous, forming a paste-like consistency. The mixture was placed in an amalgamator capsule and mixed for 5 min. The homogeneously mixed paste, of 1 mm thickness, was then transferred into a syringe and dispensed into a cylindrical mold with a height of 2 mm and a diameter of 10 mm. The mold was cured for 20 s until each layer was filled. The sample was then removed from the mold. TG CaviLiner (tgDent, London, United Kingdom) was used as a positive control.

### 2.2 Fourier transform infrared spectroscopy

**Figure 1:**
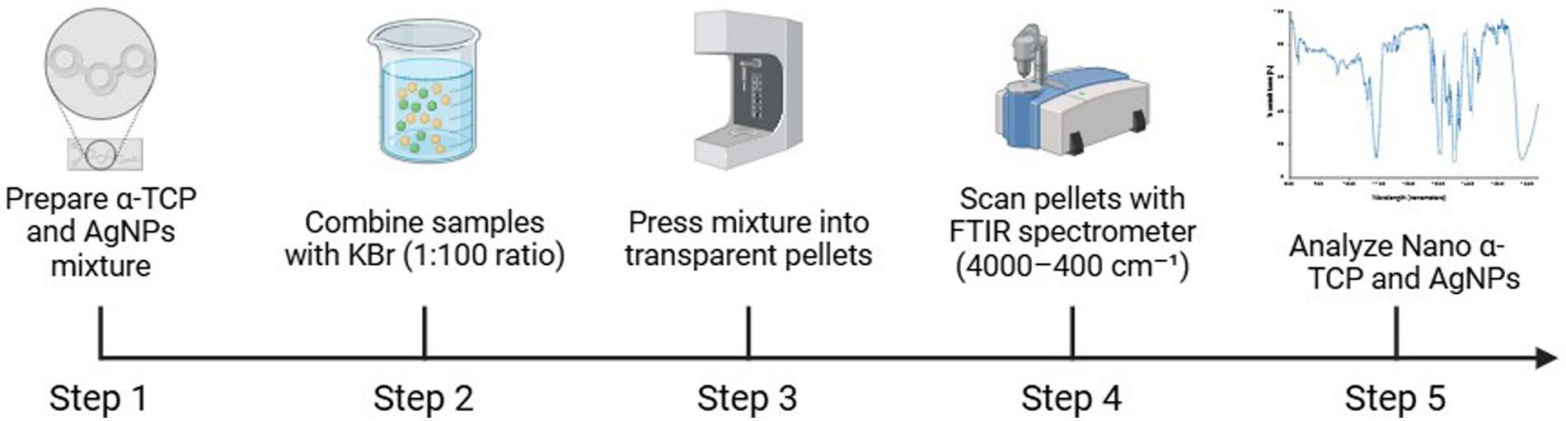
Workflow for FTIR analysis of nano α-TCP and AgNPs. Step 1: A mixture of α-tricalcium phosphate (α-TCP) and silver nanoparticles (AgNPs) was prepared. Step 2: The sample was mixed with potassium bromide (KBr) in a 1:100 ratio. Step 3: The mixture was pressed into transparent pellets. Step 4: Pellets were scanned using an FTIR spectrometer over 4,000–400 cm^−1^. Step 5: FTIR spectra were analyzed to identify functional groups and interactions in the nano α-TCP and AgNPs composite.

FTIR analysis was conducted to identify the functional groups and interactions between nano-α-TCP and AgNPs. Samples were mixed with KBr at a ratio of 1:100, pressed into transparent pellets, and scanned using an FTIR spectrometer (Shimadzu IRPrestige-21, Kyoto, Japan) in the range of 4,000–400 cm^−1^ at a resolution of 4 cm^−1^. Specific attention was directed toward phosphate (PO₄³^−^), hydroxyl (OH^−^), and carbonate (CO₃²^−^) peaks to monitor chemical bonding and matrix stability (Figure 1) (19, 20).

### 2.3 X-ray diffraction

**Figure 2:**
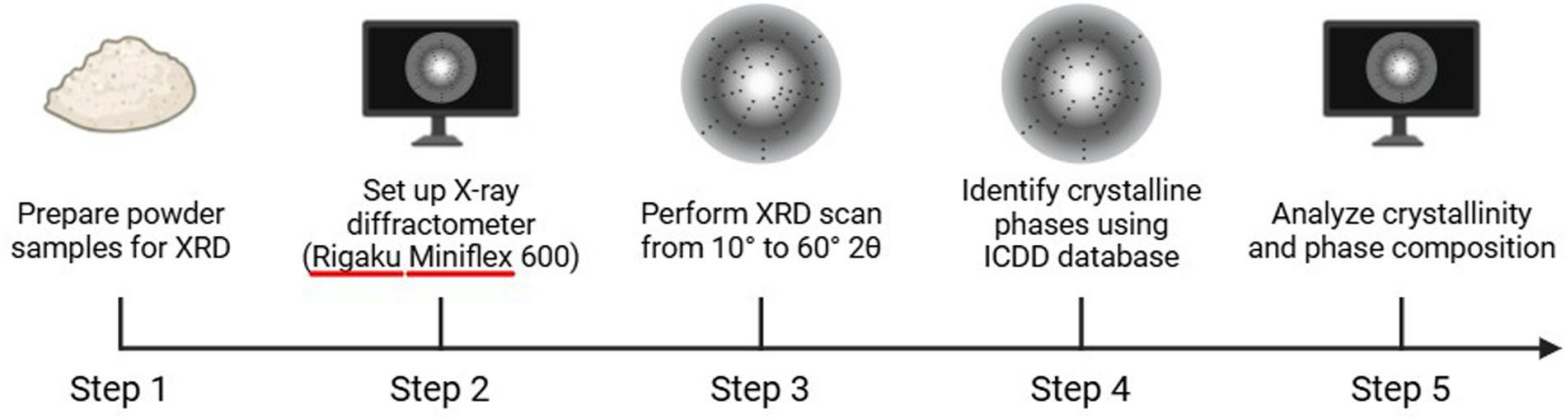
Workflow for X-ray diffraction (XRD) analysis of powdered samples. Step 1: Powder samples were prepared for XRD analysis. Step 2: The Rigaku Miniflex 600 X-ray diffractometer was set up for scanning. Step 3: XRD scanning was performed in the 2θ range of 10°–60°. Step 4: Crystalline phases were identified using the ICDD (International Centre for Diffraction Data) database. Step 5: The data were analyzed to determine crystallinity and phase composition.

XRD analysis was performed to evaluate crystallinity, phase composition, and structural changes. Powder samples were analyzed using an X-ray diffractometer (Bruker D8 Advance, Karlsruhe, Germany) with Cu-Kα radiation (*λ* = 1.5406 Å), operating at 40 kV and 30 mA. The scan range was 10°–60° 2θ at a step size of 0.02°. Crystalline phases of α-TCP, hydroxyapatite (HAp), and metallic silver (Ag) were identified using the ICDD database (Figure 2) (21).

### 2.4 SEM and energy-dispersive spectroscopy

**Figure 3:**
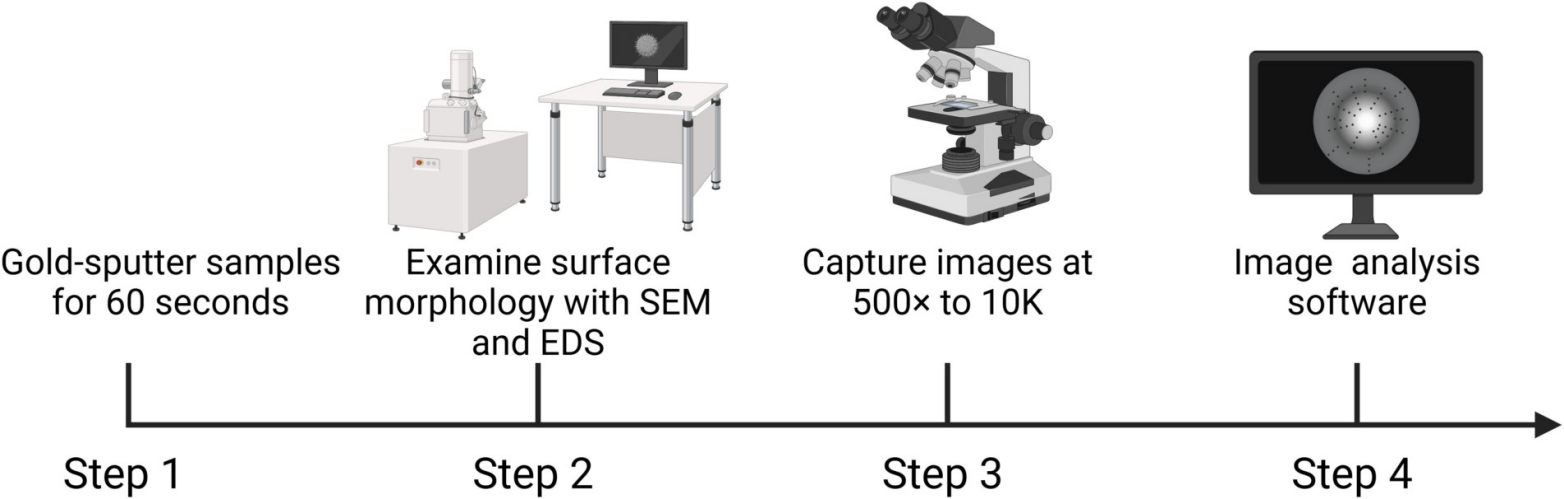
Workflow for surface morphology using scanning electron microscopy (SEM). Step 1: Samples were gold-sputtered for 60 s to enhance conductivity. Step 2: Surface morphology was examined using SEM, and elemental composition was analyzed using EDS. Step 3: Images were captured at magnifications ranging from 500× to 10,000×. Step 4: Image analysis software for SEM and EDS results.

The surface morphology was examined using SEM (SEM SU3500, Tokyo, Japan). Samples were gold-sputtered for 60 s prior to imaging. Images of the surface microstructure of the composites were captured at magnifications ranging from 500× to 10,000× (Figure 3) (22).

In addition, elemental composition was evaluated using energy-dispersive X-ray spectroscopy (EDS) attached to the SEM system (SEM-EDS SU3500, Tokyo, Japan; accelerating voltage 10 kV). EDS analysis was used to verify the Ca–P–O composition of the nano α-TCP phase and screen for other elements within the excitation volume. Since silver was incorporated as sparsely distributed nanoparticles at low local concentrations relative to the Ca–P matrix, the SEM–EDS configuration used in this study was not expected to provide precise quantitative information on Ag distribution.

### 2.5 Compressive strength testing

**Figure 4:**
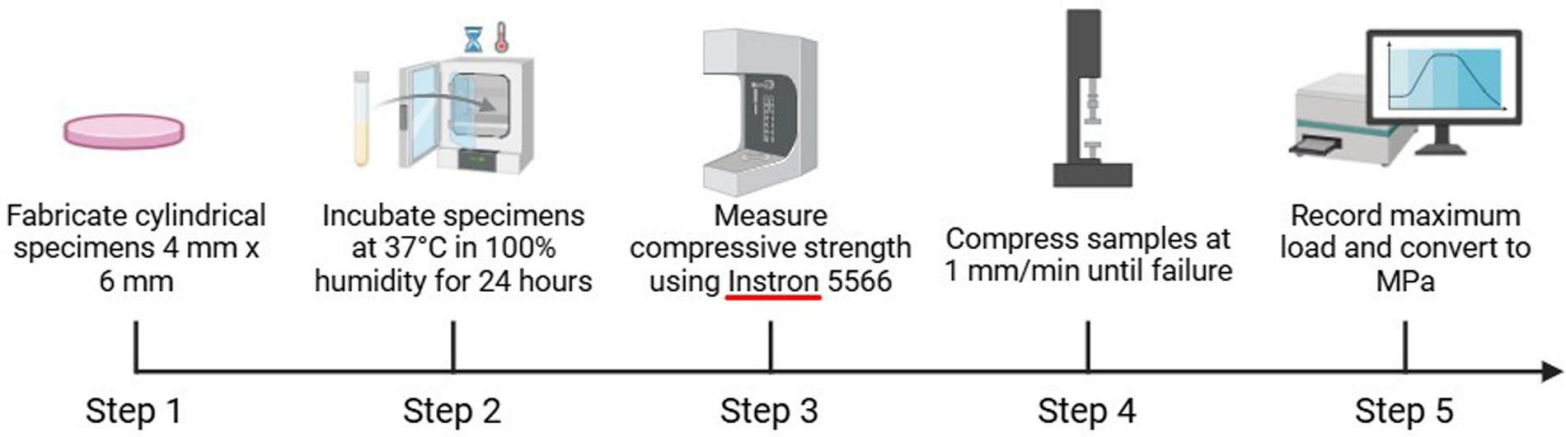
Workflow for compressive strength testing of cylindrical samples. Step 1: Cylindrical samples (4 mm × 6 mm) were fabricated. Step 2: Samples were incubated at 37 °C under 100% humidity for 24 h. Step 3: Compressive strength was measured using an Instron 5,566 universal testing machine. Step 4: Samples were compressed at a crosshead speed of 1 mm/min until failure. Step 5: The maximum load was recorded and converted to megapascals (MPa).

Compressive strength was measured using a universal testing machine (Instron 5566, Norwood, USA) in accordance with ISO 9917. Cylindrical samples (4 mm diameter × 6 mm height) were fabricated and incubated at 37 °C in 100% humidity for 24 h prior to testing. Each sample was compressed at a crosshead speed of 1 mm/min until failure. The maximum load was recorded and converted into compressive strength (MPa) (Figure 4) (23, 24).

### 2.6 pH measurement

**Figure 5:**
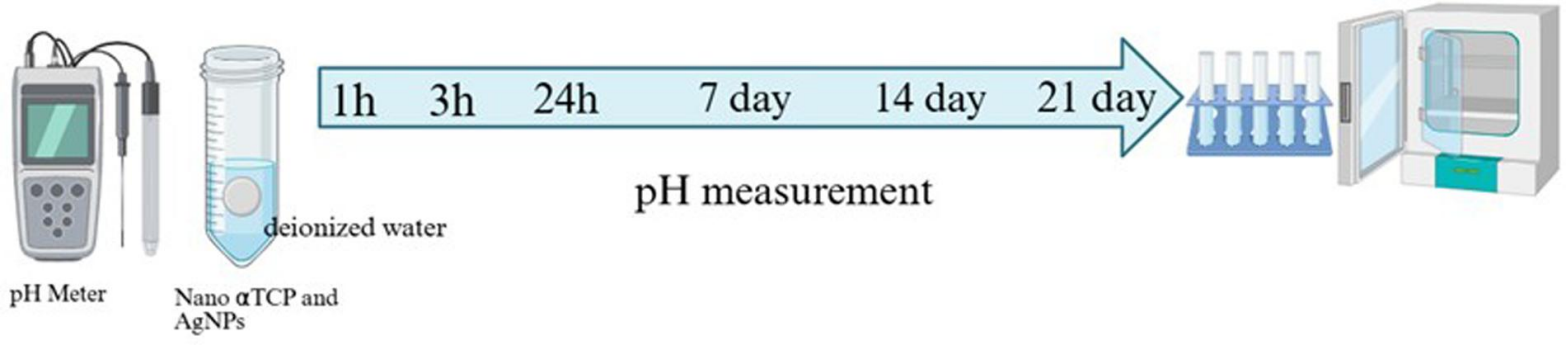
Workflow for pH measurement of nano α-TCP and AgNPs in deionized water over time. The composite was immersed in deionized water and incubated. pH was measured at multiple time points (1, 3, 24 h, 7, 14, and 21 days) using a digital pH meter to monitor the material's alkalinity profile during incubation.

The pH of the material eluates was measured to evaluate alkalinity. Disk samples (6 mm × 2 mm) were immersed in 10 mL of deionized water at 37 °C. pH values were recorded using a calibrated digital pH meter (Mettler Toledo SevenCompact™, Greifensee, Switzerland) at intervals of 1, 3, 7, and 14 days. Each measurement was performed in triplicate, and mean pH values were reported (Figure 5).

### 2.7 Calcium and phosphate ion release

To quantify ion release, samples (*n* = 3) were immersed in 10 mL of phosphate-buffered saline (PBS) at 37 °C. At predetermined intervals (1, 3, and 21 days), 1 mL of the solution was collected and replaced with fresh PBS. Calcium ion concentration was measured using Atomic Absorption Spectroscopy (AAS, PerkinElmer AAnalyst 400, Waltham, USA), while phosphate concentration was determined via UV-Vis spectrophotometry (Shimadzu UV-1800, Kyoto, Japan) using the molybdenum blue method at 650 nm. Results were expressed in mg/L (23–25).

### 2.8 Antibacterial assay

#### 2.8.1 Preparation of bacterial test suspension

The bacterial test suspension was prepared by inoculating a loopful of *Lactobacillus acidophilus* ATCC 4356 (Thermo Scientific, Massachusetts, USA) and *Streptococcus mutans* ATCC 25175 (Thermo Scientific, Massachusetts, USA) culture into Mueller-Hinton Broth (Merck, Darmstadt, Germany) and incubating at 37 °C for 24 h. Following incubation, the turbidity of the *Lactobacillus acidophilus* ATCC 4356 *Streptococcus mutans* ATCC 25175 suspension was adjusted to match a 0.5 McFarland standard. This suspension was then used for the antibacterial assay. Moreover, the total plate count (TPC) assay was performed to determine the initial bacterial population.

#### 2.8.2 Determination of initial bacterial population using the TPC method

A total of 9 mL of 0.9% NaCl was pipetted into six sterile glass vials. A decimal dilution of the bacterial suspension was performed by transferring 1 mL of the bacterial culture into the first vial containing 9 mL of 0.9% NaCl and homogenizing, resulting in a 10^−1^ dilution. Subsequently, 1 mL from the 10^−1^ dilution was transferred to the second vial and homogenized, obtaining a 10^−2^ dilution. This process was repeated until a 10^−6^ dilution was achieved in the sixth vial. In the final dilution, 1 mL was discarded to maintain the final volume. Next, 1 mL of the 10^−6^ dilution was transferred into a sterile Petri dish, followed by the addition of 20 mL of Mueller–Hinton agar (MHA). The mixture was homogenized by swirling the Petri dish in a figure-eight motion. The plates were then incubated at 37 °C for 24 h, and the colony-forming units (CFU) were counted.

#### 2.8.3 Antibacterial assay using the contact time method

The test sample was sterilized before use. The sterile sample was placed in a sterile test tube, followed by the addition of 1 mL of the bacterial suspension (adjusted to the desired absorbance). The sample was then incubated at 37 °C for 24 h. After the incubation period, a TPC assay was performed to determine the bacterial population after exposure to the test sample.

## 3 Results

### 3.1 Chemical bonding characterization (FTIR analysis)

**Figure 6:**
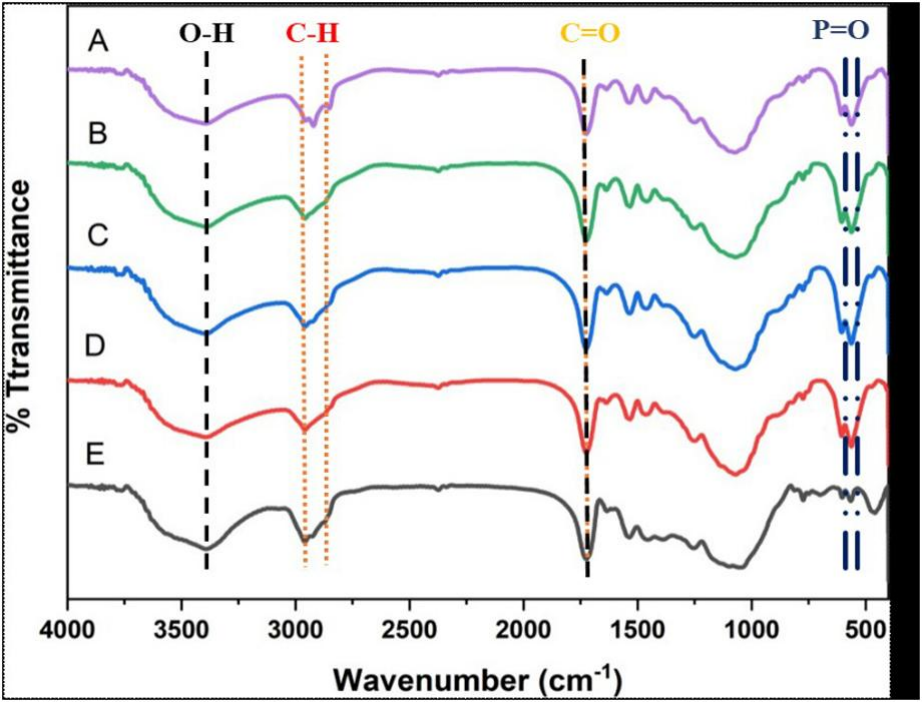
FTIR spectra of nano α-TCP and AgNPs for Samples A to E.

The functional groups in the nano α-TCP and AgNPs samples were characterized using the FTIR instrument, with the results presented in Figure 6. Transmittance is the percentage of infrared light that passes through. Wavenumber (cm^−1^) is the frequency of molecular vibrations in reciprocal centimeter units. Group A (90% nano α-TCP, 10% AgNPs) reveals peaks at 3,400 cm^−1^ (O–H from water or hydroxyl groups), 2,900 cm^−1^ (C-H from the resin matrix), 1,700 cm^−1^ (C=O), and ∼1,050 cm^−1^. The PO₄³^−^ peak at 560 cm^−1^ indicates nano α-TCP with low transmittance and high absorbance, suggesting the presence of functional groups. Group B (95% nano α-TCP, 5% AgNPs) exhibits a similar absorption pattern, although the intensity is slightly reduced; the PO₄³^−^ peak remains visible. Group C (99% nano α-TCP, 1% AgNPs) shows a consistently strong PO₄³^−^ peak, indicating the dominance of nano α-TCP. Group D (100% nano α-TCP) shows the sharpest PO₄³^−^ peak, reflecting a pure crystalline structure without AgNPs. Meanwhile, Group E (positive control) shows only the C–H and C=O peaks.

### 3.2 Crystallinity and phase composition (XRD analysis)

**Figure 7:**
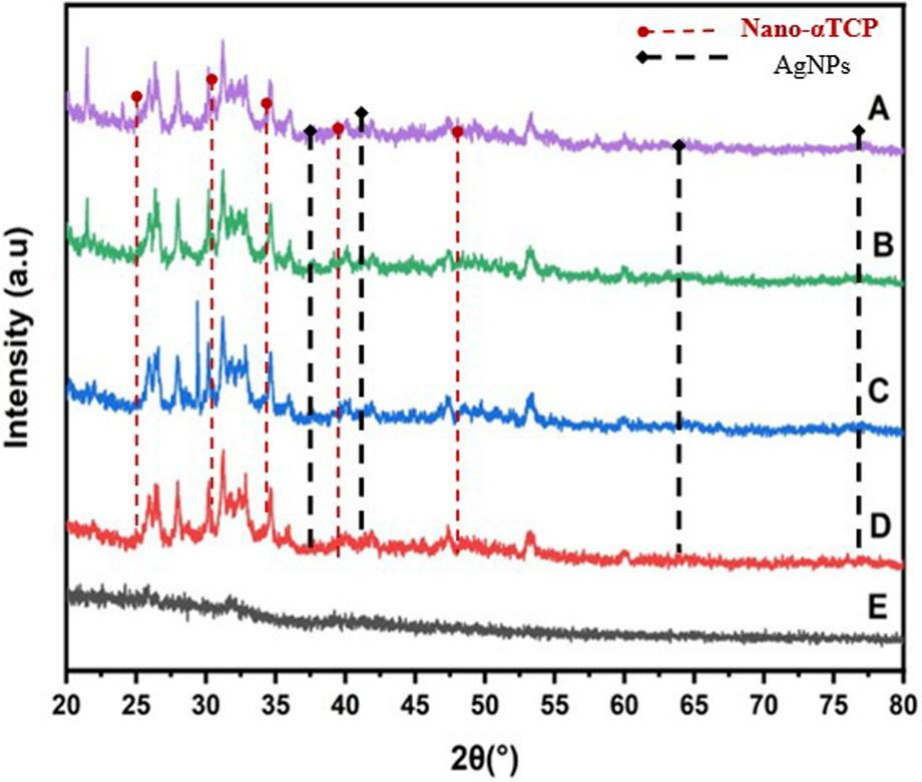
XRD spectra of the nano α-TCP and AgNPs for Samples A to E.

The crystal structure of the nano α-TCP and AgNPs samples was characterized using the XRD tool, with the results presented in Figure 7. The *X*-axis (2θ angle degrees) indicates the position of the crystal peaks, while the *Y*-axis (intensity a.u.) indicates the strength of the X-ray reflection. Group A, which contains 90% nano α-TCP and 10% AgNPs, displays characteristic peaks of nano α-TCP at angles of 31.8°, 32.9°, and 34.6°, indicating the presence of the crystalline phase of nano α-TCP. In addition, peaks at 38.1°, 44.3°, 64.4°, and 77.4° also appear, which are characteristic of AgNPs. Group B, with a concentration of 95% nano α-TCP and 5% AgNPs, exhibits nano α-TCP crystal peaks in the range of 25–35°, similar to those of Group A. The AgNPs peaks are still detected at 38.1° and 44.3°, but their intensity is lower compared to Group A, which has a higher AgNPs content (10%). This indicates that AgNPs crystals remain present, albeit in smaller quantities. Group C, with 99% nano α-TCP and 1% AgNPs, displays nano α-TCP crystal peaks at 25.8°, 31.8°, 34.6°, and 47.3°; however, the AgNPs peaks are not visible due to their very low concentration. Meanwhile, Group D, with 100% nano α-TCP without AgNPs, shows a strong and sharp crystalline pattern, indicating the typical bulk structure of nano α-TCP without interference from AgNPs particles. Group E, the control (positive), shows almost no peaks in the amorphous or very non-crystalline pattern, which is a characteristic of composite resin-based materials or organic filler materials.

### 3.3 Microstructural surface morphology and elemental composition (SEM and EDS analysis)

**Figure 8:**
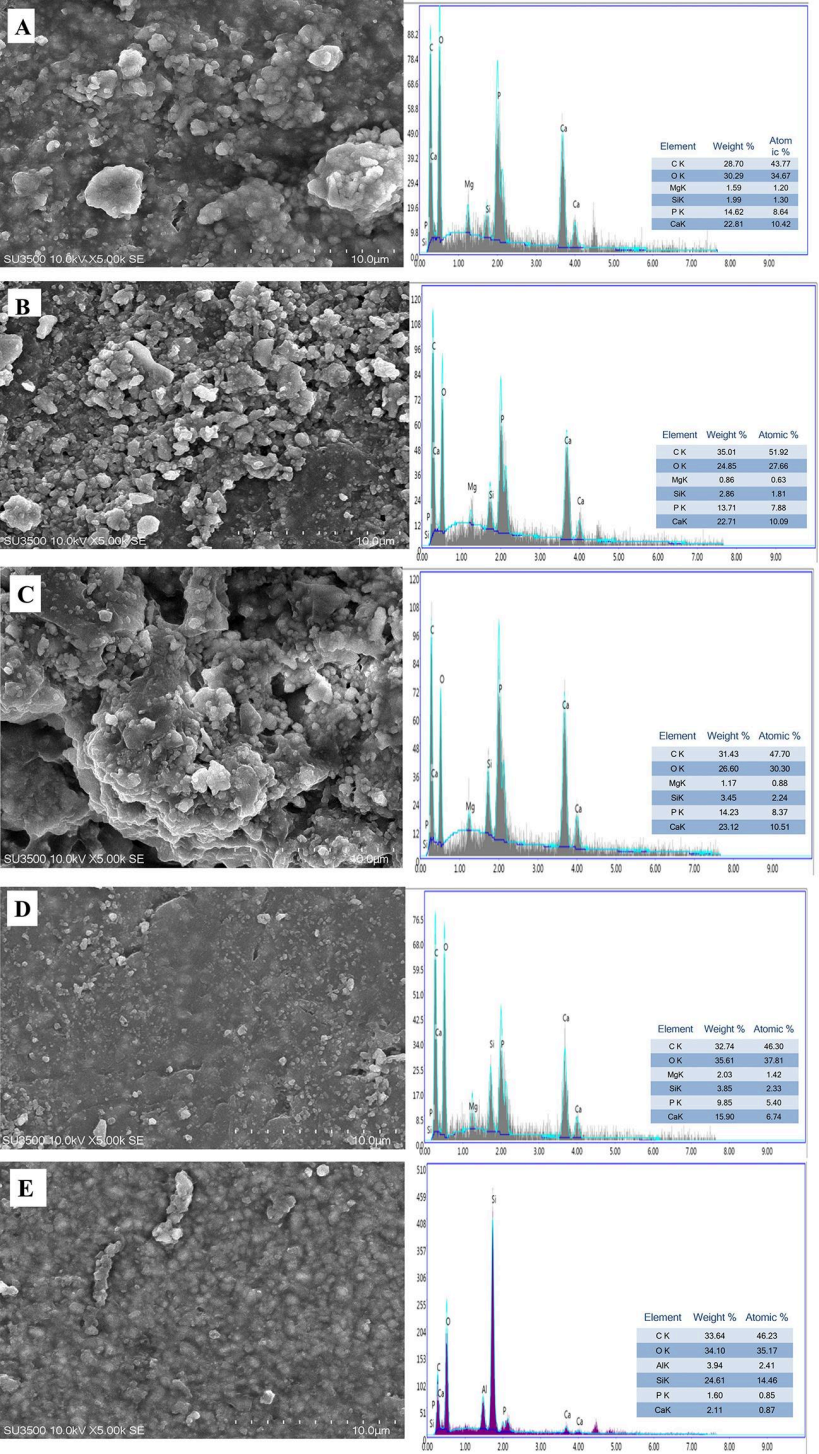
Representative SEM images of the nano α-TCP-based composites and their corresponding EDS spectra. The spectra show Ca, P, and O as the main elements, with minor Mg and Si and significant C from the resin matrix/carbon coating, confirming the Ca–P–O composition of the α-tricalcium phosphate phase. Across the analyzed regions, Ca and P contents yielded an average Ca/P weight ratio of ≈1.6, consistent with α-TCP. Distinct silver peaks were not clearly resolved, likely due to the nanoscale dispersion and the low local concentration of AgNPs relative to the Ca–P matrix; EDS was therefore primarily used to verify the Ca–P–O composition rather than to quantify Ag.

SEM micrographs revealed the morphological differences across the formulations, as shown in Figure 8. Groups A–C and D (pure nano α-TCP) demonstrated relatively fine, agglomerated particulate surfaces with increased surface roughness. Group E (control) presented a more porous architecture with larger intergranular voids, possibly facilitating ion exchange but compromising mechanical cohesion. These microstructural features are critical because they influence cellular adhesion, ion diffusion, and mechanical performance, all of which are key determinants of clinical efficacy in pulp capping. Samples A–C showed distinct agglomerated clusters (yellow arrows) of AgNPs trapped within a matrix of nano α-TCP (red arrows), forming a composite structure. In contrast, Sample D featured a smooth, even surface devoid of AgNPs agglomerates, indicating that it consists entirely of nano α-TCP. Similarly, Sample E also presented a slightly smooth surface.

SEM images of the nano α-TCP-based composites revealed an irregular, microporous surface with angular particles embedded in the resin matrix. The corresponding EDS spectra (Figure 8) confirmed Ca and P as the predominant elements, together with O and minor Mg and Si, consistent with the composition of α-TCP and the silanated filler system. Quantitative analysis from four representative regions indicated Ca contents of approximately 16–34 wt% and P contents of 14–16 wt%, yielding an average Ca/P weight ratio of about 1.6, in agreement with the expected stoichiometry of α-TCP. Distinct Ag peaks were not clearly resolved in any of the spectra, most likely because Ag was present as nanoscale clusters at low local concentrations within an excitation volume dominated by Ca and P. Therefore, in this work, EDS primarily served to confirm the Ca–P–O composition of the α-TCP phase and to exclude unexpected contaminants, rather than to quantitatively map the silver nanoparticles, which is a limitation of the technique under these conditions.

### 3.4 Alkalizing potential (pH profile)

**Figure 9:**
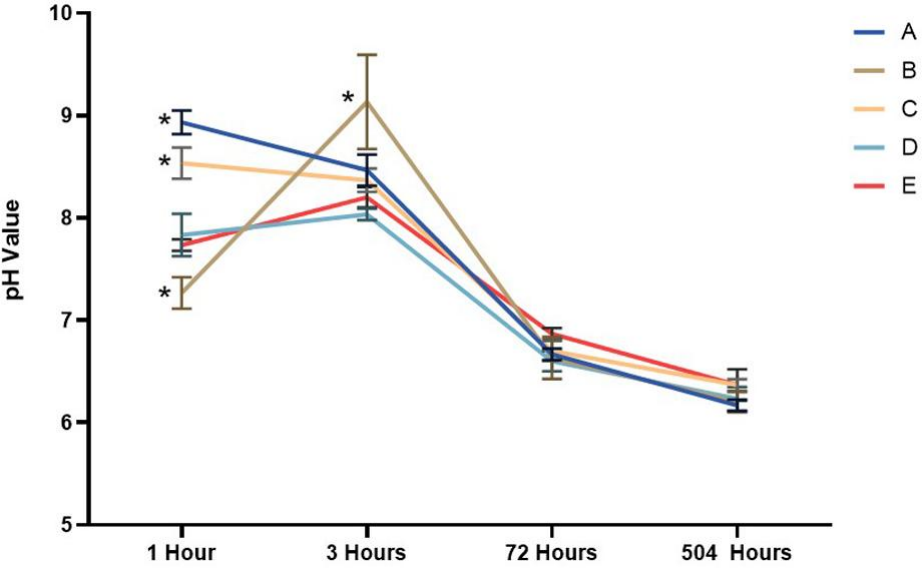
The pH changes of materials tested during the experiment. The data are expressed as mean ± SD. * indicates a significant difference compared to the positive control (*p* < 0.05) at the same time point.

Figure 9 summarizes the pH measurements taken at 1, 3, 72, and 504 h of immersion. Based on the pH test data, a Shapiro–Wilk normality test was conducted. The results indicate that the data are normally distributed (*p* < 0.05). Subsequently, a one-way ANOVA was performed, revealing a statistically significant difference (*p* < 0.05). Next, a Tukey *post-hoc* test was conducted. Measurements at the first hour showed an increase from the beginning of the test, specifically in Group A. Groups A, C, and D showed a decrease in pH values from the first hour to 504 h (21 days). Group B values increased from the first hour to the third hour, and then decreased until the 21st day. Meanwhile, Group E values also increased from the first hour to the third hour, and then decreased until the 21st day.

### 3.5 Calcium ion release

**Figure 10:**
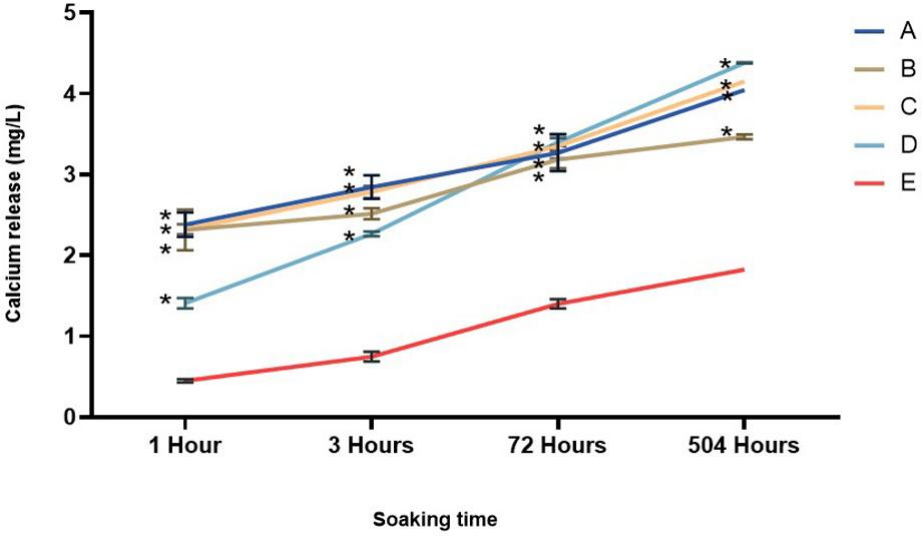
Calcium release changes of materials tested throughout the experiment. The data are expressed as mean ± SD. * indicates a significant difference compared to the positive control (*p* < 0.05) at the same time point.

The calcium release test measurements were taken at 1, 3, 72, and 504 h, as shown in Figure 10. Following the data collection from the calcium release characterization test, a normality test was performed. The Shapiro–Wilk normality test results indicated that the data followed a normal distribution (*p* > 0.05). Subsequently, a two-way ANOVA was conducted, revealing a significant difference (*p* < 0.05). In this test, all groups showed a significant difference compared to the positive control. In addition, all groups showed an increase in calcium release over time. Group A (nano α-TCP 90%, AgNPs 10%) showed stable calcium release, increasing from ∼2.1 mg/L (1 h) to ∼3.6 mg/L (504 h). Group B (nano α-TCP 95%, AgNPs 5%) showed comparable early calcium release at 1 h to Group A. By 3 h, both increased slightly (approximately 2.8–3.0 mg/L), with Group A remaining marginally higher. From 72 to 504 h, the profiles diverged: Group A continued a steeper rise, whereas Group B rose more modestly. Thus, despite similar early kinetics, Group A consistently released more calcium and achieved a higher terminal level than Group B. Group C (99% Nano α-TCP, 1% AgNPs) recorded the highest initial value (∼2.4 mg/L) and continued to increase to ∼4.3 mg/L. Meanwhile, Group D (100% nano α-TCP) showed the lowest initial release (∼1.3 mg/L) but experienced a sharp increase to ∼4.6 mg/L at 504 h, indicating a slow but continuous release of calcium ions from pure nano α-TCP. Group E (positive control) showed the lowest calcium release throughout the observation period (∼0.4–1.8 mg/L). There was a significant difference between the treatment and positive control groups at the same time (*p* < 0.05).

### 3.6 Phosphate ion release

**Figure 11:**
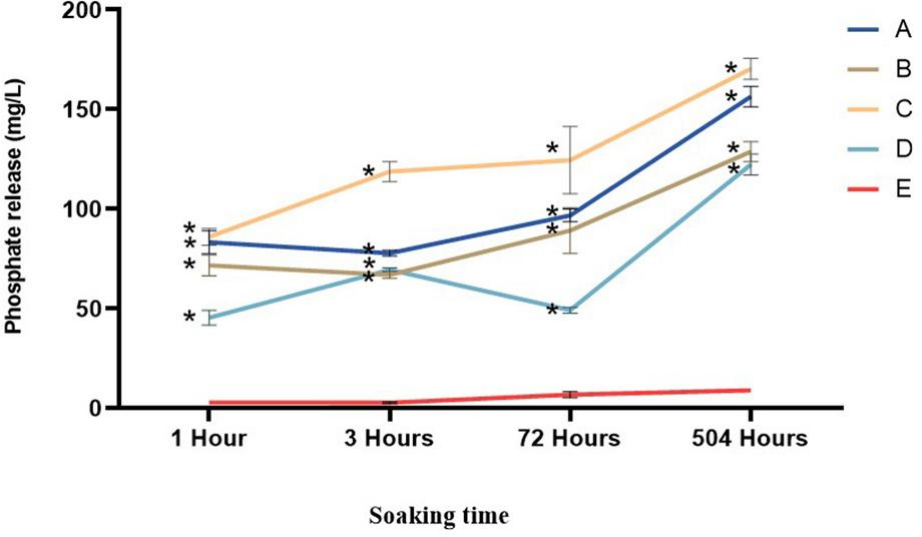
Phosphate release changes of materials tested over the time of the experiment. The data are expressed as mean ± SD. * indicates a significant difference compared to the positive control (*p* < 0.05) at the same time point.

Phosphate ion-release test measurements were conducted at 1, 3, 72, and 504 h, as illustrated in Figure 11. Based on the data obtained from the phosphate release characterization test, a normality test was performed. Subsequently, a two-way ANOVA was performed, revealing a significant difference (*p* < 0.05). In both tests, all groups showed a significant difference compared to the positive control. Moreover, all groups showed an increase in phosphate release over time. Group A (90% nano α-TCP, 10% AgNPs) showed a gradual increase in phosphate release, from ∼80 to ∼160 mg/L at 504 h. Group B (95% nano α-TCP, 5% AgNPs) followed a similar pattern but with a slightly lower final value (∼140 mg/L). Group C (99% nano α-TCP, 1% AgNPs) exhibited the highest release rate (∼170 mg/L), with a rapid initial spike at 1–3 h (∼90–120 mg/L), indicating a prompt bioactive response. Group D (100% nano α-TCP) started with a low release (∼50 mg/L at 1 h), then gradually increased to approximately 130 mg/L at 504 h, reflecting a slow, sustained phosphate release. On the other hand, the positive control group showed very low phosphate release (<10 mg/L) at all time points, indicating the absence of active phosphate.

### 3.7 Compressive strength evaluation

**Figure 12:**
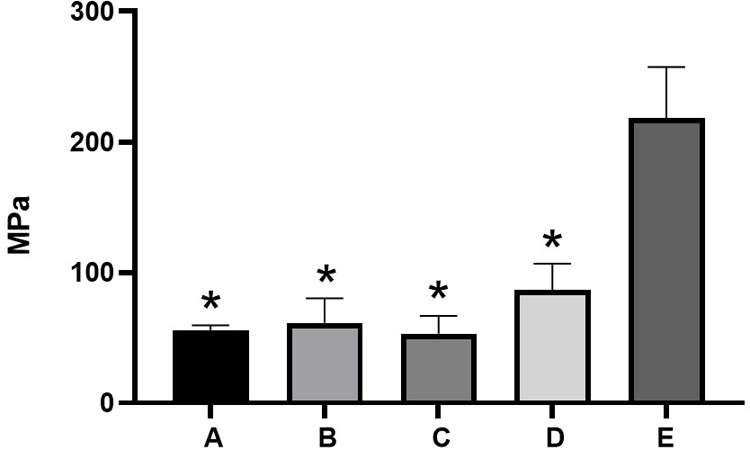
Compressive strength value of nano α-TCP and AgNPs. The data are expressed as mean ± SD. * indicates a significant difference compared to the positive control (*p* < 0.05).

Compressive testing revealed significant differences among the groups, as shown in Figure 12. Group D (pure nano α-TCP) exhibited the highest compressive strength (approximately 86 MPa), significantly higher than the AgNPs-containing groups (A–C), all of which remained below 62 MPa. Although AgNPs enhanced ion release and antibacterial potential, their addition appeared to interfere with mechanical integration within the resin matrix. This trade-off underscores the challenge in optimizing biofunctionality without compromising mechanical integrity.

### 3.8 Antibacterial activity against *L. acidophilus* and *S. mutans*

Based on the data from the antibacterial tests against *L. acidophilus* and *S. mutans* after treatment with nano-αTCP, a Shapiro–Wilk normality test was performed. The statistical results indicated a normal distribution of data for all treatment groups (*p* > 0.05). Subsequently, a one-way ANOVA test was then performed, to determine antibacterial activity against *L. acidophilus* and *S. mutans*, revealing a significant difference (*p* < 0.05).

**Figure 13:**
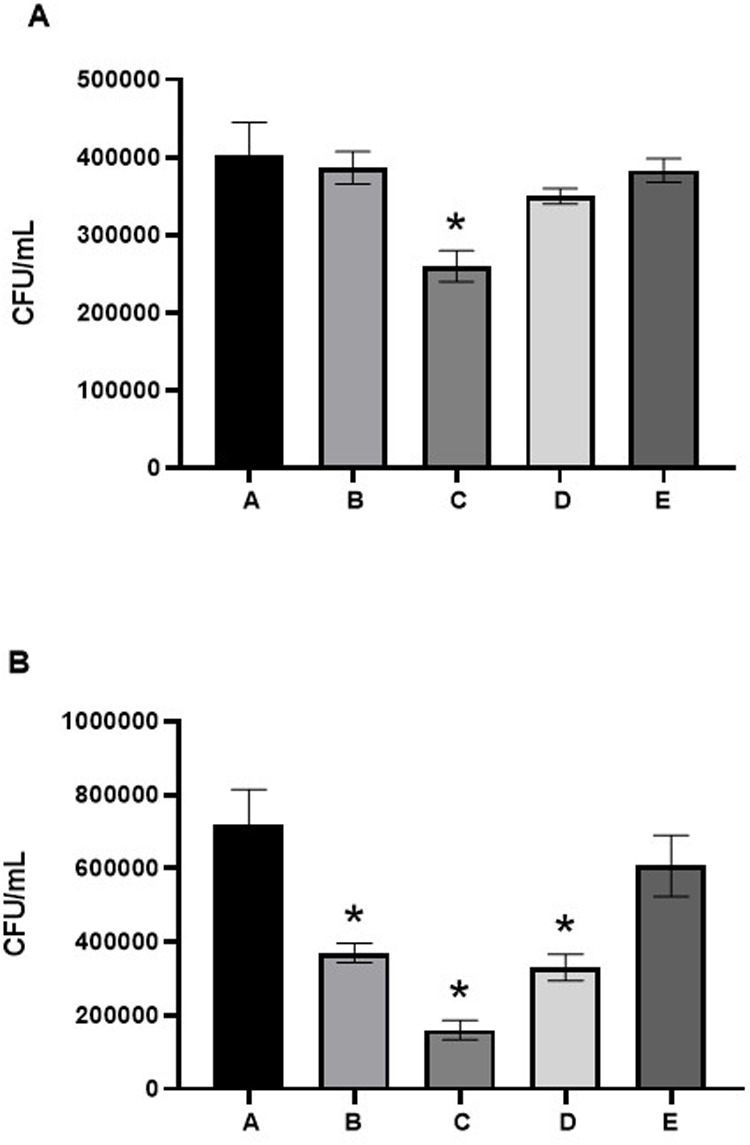
Antibacterial activity of nano α-TCP–AgNPs against **(A)** *L. acidophilus* and **(B)** *S. mutans*. Bars represent mean ± SD (*n* = 3). A one-way ANOVA followed by Tukey's *post hoc* test was used to analyze differences between groups (*p* < 0.05). Bars with different letters indicate statistically significant differences (*p* < 0.05); bars with the same letter are not significantly different.

This was followed by Tukey's *post-hoc* test, demonstrating significant differences among the experimental groups (*p* < 0.05). As shown in Figure 13, groups labeled with different letters differ significantly. Group C (1% AgNPs) displayed the strongest antibacterial activity against both *L. acidophilus* and *S. mutans*, while the control Group E exhibited the weakest inhibition. Groups A and B produced moderate effects, and Group D (pure α-TCP) showed intermediate antibacterial performance. These results confirm that incorporation of low-dose AgNPs (1%) provides optimal bactericidal efficacy without compromising material integrity.

## 4 Discussion

An effective pulp-capping substance should promote dentin regeneration, preserve pulp vitality, and exhibit strong bactericidal or bacteriostatic properties. Recent innovations in pulp capping involve combining AgNPs with nano-sized α-TCP. This dual approach is beneficial for pulp tissue, facilitating hard-tissue formation with minimal discomfort while enhancing dentin regeneration.

The physicochemical characteristics of α-TCP are crucial for its effectiveness as a dental biomaterial. Research indicates that nano-sized α-TCP exhibits greater reactivity and bioactivity than conventional forms. It promotes the effective release of Ca^2+^ and PO₄^3−^ ions, which contribute to the formation of hydroxyapatite layers essential for remineralization, especially important in pulp-capping applications (8). This ion-exchange capability has been validated in previous studies, demonstrating that α-TCP can enhance bond strength in dentin adhesives when utilized as a filler (26). Morphological analyses using SEM reveal that the irregular shapes and agglomeration of α-TCP nanoparticles positively influence mechanical properties (27).

FTIR spectra of nano α-TCP and AgNPs composites reveal important functional groups that contribute to mineralization and bioactivity, specifically calcium oxides and phosphate. Notably, Sample E displays a strong Ca-O vibration at approximately 459 cm^−1^, indicating the presence of bulk calcium phosphate rather than nano α-TCP. This distinction may influence its bioactivity and absorption properties (28). Nano-sized materials, such as bioactive glass and calcium phosphates, possess a higher surface area and reactivity, enabling more effective responses to biological stimuli. Phosphate groups play a crucial role in mineral tissue formation, aligning with previous studies that highlight the potential of nano calcium phosphate to promote bone growth (29).

The XRD measurements indicated that formulations A–D contained a significant amount of crystalline nano α-TCP, which is essential for pulp therapy due to its stable structure. The incorporation of AgNPs at 1%–10% concentrations did not alter the crystal phase of nano α-TCP, enabling the antibacterial properties to help control infections without compromising bioactivity. High levels of Ag in Group A were effective at killing bacteria but raised concerns about potential cellular harm. In contrast, moderate amounts of Ag in Groups B and C provided a balanced functional profile. Meanwhile, Group D, consisting of pure nano α-TCP, retained its ability to mineralize completely and continued to combat bacteria effectively (30).

SEM analysis revealed a porosity of 57.64%, which falls within the optimal range for bioactive scaffolds. The interconnected pore structure is crucial for pulp tissue regeneration because it facilitates ion movement and enables cells to migrate into and out of the material matrix. High porosity also increases the active surface area, promoting the initial steps of hydroxyapatite crystal formation and subsequent accumulation of these crystals to develop new mineralized tissue. Both processes are necessary for remineralization and bonding with dentin, ensuring physiological and functional integration with tooth structure (31, 32).

In pulp-capping applications, the bioactive and antibacterial properties of highly porous materials are considered more crucial than their mechanical strength for facilitating healing. Nano α-TCP may release calcium and phosphate ions, while AgNPs exhibit long-lasting antibacterial effects. Together, these two materials help the body heal while also preventing secondary infections. Therefore, the SEM results in this study provide evidence that the nano α-TCP and AgNPs formulation exhibits the best microstructural properties for use as a pulp-capping material. There are numerous tiny pores on the material's surface, increasing its surface area and accelerating ion release, with particles evenly distributed throughout. These characteristics make this substance an excellent candidate for dental treatments aimed at preserving pulp vitality and encouraging natural healing, aligning with the latest approaches in dental care that emphasize tissue regeneration (33).

All nano-α-TCP and AgNPs composites increased pulp alkalinity, which is essential for promoting healing and inhibiting bacterial growth. Group B achieved a pH level of 9.2, facilitating the release of numerous ions. After 3 h, the pH levels across all groups stabilized at approximately 6.2 and remained consistent for 21 days. This stability indicates ongoing ion release, which is advantageous for mineralization and cell viability (30, 34). Interestingly, the composite containing the highest AgNPs fraction (10%; Group A) exhibited weaker antibacterial activity than the 1% AgNPs formulation (Group C). This can be explained by the microstructural behavior of highly filled nanocomposites: At elevated silver content, nanoparticles tend to agglomerate into larger clusters and become more deeply embedded in the resin matrix, reducing the available specific surface area for bacterial contact and limiting Ag^+^ diffusion into the surrounding medium. In contrast, a low AgNPs loading (1%) favors a more homogeneous dispersion, as also supported by the SEM-EDS images, allowing a greater proportion of particles to remain accessible at or near the composite surface. This results in more efficient Ag^+^ ion release and more direct interaction with bacterial cells, leading to the strongest bactericidal effect despite the lower nominal silver content. Similar nonlinear dose–response relationships have been reported for AgNPs-containing dental composites, where excessive nanoparticle loading leads to agglomeration and diminished antimicrobial efficacy (33–35). From a caries management perspective, the ability of the nano α-TCP/AgNPs composites to inhibit cariogenic species while maintaining a remineralizing, alkaline microenvironment suggests that these materials could be integrated into minimally invasive strategies for treating deep carious lesions, where preservation of pulp vitality is the central objective. The Ca^2+^ and PO₄^3−^ ions were released over an extended period, particularly in Group C, indicating sustained bioactivity. The Ca/P ratio of approximately 1.6 is beneficial for odontoblast growth and hydroxyapatite formation. This makes it superior to standard calcium hydroxide materials, as it releases more ions when indicator levels such as ALP and DSPP are elevated (34). The positive control group demonstrated the highest compressive strength, while all experimental groups (A–D) had lower compressive strength. However, they still met the ISO 3107 minimum requirement of 5 MPa. Among all the nano α-TCP formulations, Group C exhibited the best mechanical performance. For pulp capping to be effective, the material must possess both mechanical strength and biological properties, including bioactivity, regenerative potential, and antibacterial action. Future enhancements could involve incorporating glass ionomer or biodentine-like matrices to further improve mechanical strength (34, 35).

The results of the antibacterial test against *L. acidophilus* showed that Group C had the highest bactericidal activity, followed by Group B, while Group A showed the weakest bactericidal effect*.* Significant differences were observed between Groups A and C, with Group C also showing significant differences compared to the positive control. The same trend was observed in antibacterial tests against *S. mutans*, with Group C showing the highest bactericidal activity. This study shows that nano α-TCP and 1% AgNPs are the most potent formula for killing *L. acidophilus* and *S. mutans.* The low concentration of 1% AgNPs (Group C) actually provided better particle dispersion, preventing clumping and resulting in optimal Ag^+^ ion release, more effective direct interaction with bacteria, and a more stable, enhanced antibacterial effect (36–38).

Silver nanoparticles (AgNPs) exhibit potent antimicrobial activity against *L. acidophilus* and *S. mutans* through multiple mechanisms involving cell membrane disruption, oxidative stress, and the release of silver ions (Ag^+^). Electrostatic interactions between positively charged AgNPs and negatively charged bacterial cell surface cause structural damage to the membrane, leakage of cell contents, and cell death (39). Furthermore, AgNPs induce the formation of ROS that damage proteins, lipids, and DNA, releasing Ag^+^ ions that bind to the thiol groups of essential enzymes, thereby inhibiting bacterial cell respiration and replication. Their effectiveness against *L. acidophilus* and *S. mutans* is due to their small particle size (5–50 nm), which enables penetration of the thick peptidoglycan layer characteristic of gram-positive bacteria, as well as their ability to prevent biofilm formation and acid production (40). The bactericidal properties of AgNPs stem from their high surface area, continuous ion release, and ability to produce oxidative stress, leading to synergistic effects on various cellular targets (41, 42).

The antibacterial and physicochemical tests in this study were conducted *in vitro*, indicating that the results may not fully represent the complex biological environment of human pulp tissue. Furthermore, the biocompatibility of nano-α-TCP and AgNPs composites has not been assessed in cellular or animal models, which is essential for establishing the clinical relevance of the findings. Future studies should prioritize *in vivo* evaluations, including cytotoxicity, histological pulp response, and dentin bridge formation, to assess the long-term safety and efficacy of this dual-functional material in vital pulp therapy. In addition, future work should focus on enhancing the resin matrix to improve its strength while maintaining its ion-releasing capabilities.

## 5 Conclusion

This study demonstrated that the nano α-TCP/AgNPs composites exhibit favorable physicochemical properties for potential application as pulp-capping materials. The biocomposites maintained a crystalline α-TCP structure, generated an alkaline environment, and released calcium and phosphate ions in a sustained manner. In addition, they displayed effective antibacterial activity, particularly in formulations containing low concentrations of AgNPs (1%). Although higher AgNPs content slightly reduced compressive strength, all formulations remained within the acceptable range as defined by ISO standards. These findings indicate that nano α-TCP/AgNPs composites possess promising characteristics. However, their biological responses and regenerative potential should be further verified through *in vitro* and *in vivo* studies.

## Data Availability

The original contributions presented in the study are included in the article/Supplementary Material, further inquiries can be directed to the corresponding authors.

## References

[1] Leye Benoist F Gaye Ndiaye F Kane AW Benoist HM Farge P. Evaluation of mineral trioxide aggregate (MTA) versus calcium hydroxide cement (Dycal((R))) in the formation of a dentine bridge: a randomised controlled trial. Int Dent J. (2012) 62:33–9. 10.1111/j.1875-595X.2011.00084.x 22251035 PMC9374926

[2] Islam R Islam MRR Tanaka T Alam MK Ahmed HMA Sano H. Direct pulp capping procedures—evidence and practice. Jpn Dent Sci Rev. (2023) 59:48–61. 10.1016/j.jdsr.2023.02.002 36880059 PMC9985044

[3] Cahyanto A Rezano A Zakaria MN El-Ghannam A. Synthesis and characterization of a novel SCPC-CO3Ap cement for pulp capping application in dentistry. Key Eng Mater. (2017) 758:29–33. 10.4028/www.scientific.net/KEM.758.29

[4] Lee JB Park SJ Kim HH Kwon YS Lee KW Min KS. Physical properties and biological/odontogenic effects of an experimentally developed fast-setting α-tricalcium phosphate-based pulp capping material. BMC Oral Health. (2014) 14:87. 10.1186/1472-6831-14-87 25015173 PMC4105101

[5] Fosca MA-O Streza A Antoniac IA-O Vadalà G Rau JA-O. Ion-Doped calcium phosphate-based coatings with antibacterial properties. J Funct Biomater. (2023) 14(5):250. 10.3390/jfb14050250 37233360 PMC10219342

[6] Canillas M Pena P de Aza AH Rodríguez MA. Calcium phosphates for biomedical applications. Bol Soc Esp Cerám Vidr. (2017) 56:91–112. 10.1016/j.bsecv.2017.05.001

[7] Tronco MC Cassel JB dos Santos LA. α-TCP-based calcium phosphate cements: a critical review. Acta Biomater. (2022) 151:70–87. 10.1016/j.actbio.2022.08.040 36028195

[8] Vecbiškena L Gross K-A Riekstiņa U Yang TC. Crystallized nano-sized alpha-tricalcium phosphate from amorphous calcium phosphate: microstructure, cementation and cell response. Biomed Mater. (2015) 10:025009. 10.1088/1748-6041/10/2/025009 25886478

[9] Ballal NV Shavi GV Kumar R Kundabala M Bhat KS. *In vitro* sustained release of calcium ions and pH maintenance from different vehicles containing calcium hydroxide. J Endod. (2010) 36:862–6. 10.1016/j.joen.2009.12.021 20416434

[10] Xu H Weir M Sun L. Calcium and phosphate ion releasing composite: effect of pH on release and mechanical properties. Dent Mater. (2008) 25:535–42. 10.1016/j.dental.2008.10.009 19101026 PMC2649691

[11] Bee S-L Bustami Y Ul-Hamid A Lim K Abdul Hamid ZA. Synthesis of silver nanoparticle-decorated hydroxyapatite nanocomposite with combined bioactivity and antibacterial properties. J Mater Sci: Mater Med. (2021) 32:106. 10.1007/s10856-021-06590-y 34426879 PMC8382650

[12] Konappa N Patil RH Kariyappa AS Krishnamurthy S Ramachandrappa NS Krishnappa R Green synthesis of silver nanoparticles using *Amomum nilgiricum* leaf extracts: preparation, physicochemical characterization and ameliorative effect against human cancer cell lines. Cytotechnology. (2025) 77(1):16. 10.21203/rs.3.rs-5197419/v1 39669689 PMC11631834

[13] Zhang X Liu Z-g Shen W Gurunathan S. Silver nanoparticles: synthesis, characterization, properties, applications, and therapeutic approaches. Int J Mol Sci. (2016) 17:1534. 10.3390/ijms17091534 27649147 PMC5037809

[14] Afkhami F Forghan P Gutmann JL Kishen A. Silver nanoparticles and their therapeutic applications in endodontics: a narrative review. Pharmaceutics. (2023) 15(3):715. 10.3390/pharmaceutics15030715 36986576 PMC10052550

[15] Toida Y Kawano S Islam R Jiale F Chowdhury AA Hoshika S Pulpal response to mineral trioxide aggregate containing phosphorylated pullulan-based capping material. Dent Mater J. (2022) 41:126–33. 10.4012/dmj.2021-153 34602588

[16] Sari AF Nirwana I Yuliati A Meizarini A Rahayu RP Palupi R Alexandra MF Nuraida TB Surboyo MD Shariff KA Anti-inflammatory effects of calcium hydroxide combined with ellagic acid as pulp capping material: *in vivo* study. Eur J Dent. (2024) 19(3):624–910. 10.1055/s-0044-1791243 39510525 PMC12182428

[17] Bakır EP Yıldırım ZS Bakır Ş Ketani A. Are resin-containing pulp capping materials as reliable as traditional ones in terms of local and systemic biological effects? Dent Mater J. (2022) 41:78–86. 10.4012/dmj.2021-065 34483201

[18] Ferracane JL. Resin composite—state of the art. Dent Mater. (2011) 27:29–38. 10.1016/j.dental.2010.10.020 21093034

[19] Valentim RM Andrade SM Dos Santos ME Santos AC Pereira VS Dos Santos IP Composite based on biphasic calcium phosphate (HA/β-TCP) and nanocellulose from the açaí tegument. Materials (Basel). (2018) 11(11):2213. 10.3390/ma11112213 30412992 PMC6266682

[20] da Fonseca SC Freitas RB Sotiles AR Schemczssen-Graeff Z Miranda IM Biscaia SM 3D Scaffold of hydroxyapatite/*β* tricalcium phosphate from mussel shells: synthesis, characterization and cytotoxicity. Heliyon. (2025) 11:e41585. 10.1016/j.heliyon.2024.e41585 39866499 PMC11758959

[21] Madhukumar K Varma H Komath M Elias T Padmanabhan V Nair C. Photoluminescence and thermoluminescence properties of tricalcium phosphate phosphors doped with dysprosium and europium. Bull Mater Sci. (2007) 30:527–34. 10.1007/s12034-007-0082-x

[22] Karydis-Messinis A Moschovas D Markou M Tsirka K Gioti C Bagli E Hydrogel membranes from chitosan-fish gelatin-glycerol for biomedical applications: chondroitin sulfate incorporation effect in membrane properties. Gels. (2023) 9(11):844. 10.3390/gels9110844 37998934 PMC10670475

[23] Hamdy TM. Effect of E-glass fibers addition on compressive strength, flexural strength, hardness, and solubility of glass ionomer based cement. BMC Oral Health. (2024) 24:739. 10.1186/s12903-024-04447-8 38937723 PMC11210041

[24] Zakaria MN Cahyanto A El-Ghannam A. Calcium release and physical properties of modified carbonate apatite cement as pulp capping agent in dental application. Biomater Res. (2018) 22:35. 10.1186/s40824-018-0146-6 30546914 PMC6282351

[25] Kang S. Mineralization-inducing potentials of calcium silicate-based pulp capping materials in human dental pulp cells. Yeungnam Univ J Med. (2020) 37:217–25. 10.12701/yujm.2020.00248 32438533 PMC7384909

[26] Wang S Wang Y Sun K Sun X. Low temperature preparation of *Α*-tricalcium phosphate and its mechanical properties. Process Appl Ceram. (2017) 11:100–5. 10.2298/PAC1702100W

[27] Al-Qahtani AS Tulbah HI Binhasan M Shabib S Al-Aali KA Alhamdan MM Influence of concentration levels of *Β*-tricalcium phosphate on the physical properties of a dental adhesive. Nanomaterials. (2022) 12:853. 10.3390/nano12050853 35269344 PMC8912458

[28] Baskaran V Madhubala M Menon T Gopal S Venkatesan S. Synthesis and characterization of nisin-incorporated alpha-tricalcium phosphate for pulp capping—an *in vitro* study. Endodontology. (2022) 34:282. 10.4103/endo.endo_114_22

[29] Pasieczna-Patkowska S Cichy M Flieger J. Application of Fourier transform infrared (FTIR) spectroscopy in characterization of green synthesized nanoparticles. Molecules. (2025) 30(3):684. 10.3390/molecules30030684 39942788 PMC11821210

[30] Mierzejewska ŻA Rusztyn B Łukaszuk K Borys J Borowska M Antonowicz B. The latest advances in the use of nanoparticles in endodontics. Appl Sci. (2024) 14(17):7912. 10.3390/app14177912

[31] Priyadharshini SA-OX Ragavendran CA-O Sherwood AA-O Ramya JA-O Krithikadatta JA-OX. Evaluation of mineral induction ability and cytotoxicity of carbonated hydroxyapatite for pulp tissue regeneration: an *in vitro* study. Restor Dent Endod. (2024) 49(4):e40. 10.5395/rde.2024.49.e40 39649530 PMC11621306

[32] Souza AP Neves JG Navarro da Rocha D Lopes CC Moraes ÂM Correr-Sobrinho L Chitosan/Xanthan/hydroxyapatite-graphene oxide porous scaffold associated with mesenchymal stem cells for dentin-pulp complex regeneration. J Biomater Appl. (2023) 37:1605–16. 10.1177/08853282231155570 36740600

[33] Turnbull G Clarke J Picard F Riches P Jia L Han F Li B Shu W 3D bioactive composite scaffolds for bone tissue engineering. Bioact Mater. (2018) 3(3):278–314. 10.1016/j.bioactmat.2017.10.001 29744467 PMC5935790

[34] Guerrero-Gironés J Alcaina-Lorente A Ortiz-Ruiz C Ortiz-Ruiz E Pecci-Lloret MP Ortiz-Ruiz AJ Biocompatibility of a HA/β-TCP/C scaffoldas a pulp-capping agent for vital pulp treatment: an *in vivo* study in rat molars. Int J Environ Res Public Health. (2021) 18:3936. 10.3390/ijerph18083936 33918101 PMC8068992

[35] Tuygunov N Zakaria MN Yahya NA Abdul Aziz A Cahyanto A. Efficacy and bone-contact biocompatibility of glass ionomer cement as a biomaterial for bone regeneration: a systematic review. J Mech Behav Biomed Mater. (2023) 146:106099. 10.1016/j.jmbbm.2023.106099 37660446

[36] Kasraei S Sami L Hendi S Alikhani MY Rezaei-Soufi L Khamverdi Z. Antibacterial properties of composite resins incorporating silver and zinc oxide nanoparticles on *Streptococcus mutans* and Lactobacillus. Restor Dent Endod. (2014) 39:109–14. 10.5395/rde.2014.39.2.109 24790923 PMC3978100

[37] Correa JM Mori M Sanches HL da Cruz AD Poiate E Poiate J Silver nanoparticles in dental biomaterials. Int J Biomater. (2015) 2015:485275. 10.1155/2015/485275 25667594 PMC4312639

[38] Gimenez-Ingalaturre AC Rubio E Chueca P Laborda F Goñi P. Contribution to optimization and standardization of antibacterial assays with silver nanoparticles: the culture medium and their aggregation. J Microbiol Methods. (2022) 203:106618. 10.1016/j.mimet.2022.106618 36368469

[39] Ghabban H Alnomasy SF Almohammed H Al Idriss OM Rabea S Eltahir Y. Antibacterial, cytotoxic, and cellular mechanisms of green synthesized silver nanoparticles against some cariogenic bacteria (*Streptococcus mutans* and *Actinomyces viscosus*). J Nanomater. (2022) 2022:9721736. 10.1155/2022/9721736

[40] Mohammed ABA Hegazy AE Salah A. Novelty of synergistic and cytotoxicity activities of silver nanoparticles produced by Lactobacillus acidophilus. Appl Nanosci. (2023) 13:633–40. 10.1007/s13204-021-01878-5

[41] Butrón-Téllez Girón C De Alba-Montero I Hernández-Arteaga LO Garrocho-Rangel A Ruiz F. Antibacterial effect, antiadherence, and antiacidogenicity properties of a dental varnish containing silver nanoparticles: an *in vitro* study. Eur Arch Paediatr Dent. (2025) 26:799–809. 10.1007/s40368-025-01048-z 40335847

[42] Panpaliya NP Dahake PT Kale YJ Dadpe MV Kendre SB Siddiqi AG *In vitro* evaluation of antimicrobial property of silver nanoparticles and chlorhexidine against five different oral pathogenic bacteria. Saudi Dent J. (2019) 31:76–83. 10.1016/j.sdentj.2018.10.004 30723364 PMC6349994

